# Data on behavioural intention to use AI copilot through TAM and AI ecological education policy lens

**DOI:** 10.1016/j.dib.2025.111686

**Published:** 2025-05-18

**Authors:** Emelie C. Villaceran, Celbert M. Himang

**Affiliations:** a College of Computer, Information and Communications Technology, Cebu Technological University, M.J. Cuenco Ave cor. R. Palma St., Cebu City, Philippines; b Graduate School, Cebu Technological University, M.J. Cuenco Ave., cor R. Palma St., Cebu City, Philippines

**Keywords:** AI copilot, AI ecological policy framework, Technology acceptance model, Structural equation modeling, AI in education

## Abstract

This research presents a dataset concerning the behavioral intention to utilize AI Copilot among faculty and students at Cebu Technological University, analyzed through the lenses of the Technology Acceptance Model (TAM) and the AI Ecological Education Policy Framework. Data collection was conducted via a quantitative survey distributed electronically, targeting a broad participant that included various students and instructors from multiple academic departments and year levels. A non-probability convenience sampling method was employed, targeting students and instructors within CTU. The survey was meticulously designed to evaluate constructs aligned with the Technology Acceptance Model and the pedagogical components of the AI Ecological Policy Framework, emphasizing aspects such as perceived usefulness and ease of use. Informed consent was obtained from participants, ensuring an understanding of the study’s objectives and their rights regarding confidentiality. Ultimately, 414 responses were collected, with 18 responses excluded due to a lack of variability. The dataset, predominantly featuring student responses (94 %) and a smaller faculty representation (6 %), offers insights pertinent to AI Copilot's acceptance in this academic context. This dataset boasts considerable potential for future research endeavours, including studies on technology adoption in educational institutions, comparative analyses between different settings, and the enhancement of AI tools aligned with educational practices. Researchers and policymakers can utilize these insights to inform strategies for effective AI integration in education.

Specifications Table

**Table:** 

Subject	Social Sciences
Specific subject area	Educational Management, Educational Technology, AI in Education
Type of data	Table, Figure, Raw, Analyzed, Filtered, Processed etc.
Data collection	Data were collected using an online survey designed to assess perceptions of AI Copilot adoption among respondents at Cebu Technological University. The survey included items related to the Technology Acceptance Model(TAM) and AI Ecological Education Policy Framework(AIEEPF), using a 5-point Likert scale. A total of 414 responses were initially gathered, with 18 respondents excluded due to low variability in their answers, resulting in 396 valid responses. Data normalization involved assessing reliability through Cronbach’s alpha. Question items were derived from existing literature on technology acceptance and educational frameworks.
Data source location	The data for the study were collected at Cebu Technological University, which is in Cebu City, Philippines. Cebu serves as the capital of Cebu Province and is situated in the Central Visayas of the Philippines with approximately 10.3157o N latitude and 123.8854o longitude.
Data accessibility	Repository name: mendeley.com Data identification number: 10.17632/nmtc4m67d7.3 Direct URL to data: Villaceran, Emelie (2025), “Data on Behavioural Intention to Use AI Copilot Through TAM and AI Ecological Policy Lens”, Mendeley Data, V3, doi: 10.17632/nmtc4m67d7.3 It can be accessed through this link https://data.mendeley.com/datasets/nmtc4m67d7/3
Related research article	none

## 1 Value of the Data

• The data provides insights into the positive perceptions of AI Copilot, guiding educators and administrators in technology adoption decisions. The findings on skills like critical thinking and problem-solving inform curriculum development to better prepare the students for the workplace. Emphasizing stakeholder engagement fosters collaboration and shared responsibility in implementing AI technologies, ensuring ethical considerations. The holistic understanding helps create an effective educational environment that leverages AI for enhanced learning experiences while promoting responsible use and alignment with societal values. Ultimately, the data supports informed decision-making, curriculum relevance, and community involvement in educational practices, enhancing overall educational quality and effectiveness.
• Other researchers can reuse the data by conducting comparative studies to analyze AI Copilot adoption across different educational institutions or demographics, thereby identifying trends and variations in technology acceptance. The demographic profiles of respondents can support segmentation analyses, allowing researchers to examine specific behaviors and attitudes among various groups, such as age or year level. Additionally, the data can serve as a benchmark for longitudinal studies, enabling the exploration of how perceptions of AI in education change over time. It can also be applied to assess the effectiveness of AI tools in relation to pedagogical outcomes, fostering insights into best practices. Furthermore, the findings can inform the development of new research instruments, ensuring validity and reliability in measuring technology acceptance. By integrating this dataset into broader frameworks, researchers can enhance understanding of the factors influencing AI adoption in educational contexts.
• Besides the inherent value of the data, researchers can reuse it to validate or challenge existing theories on technology adoption in education, prompting deeper discussions on pedagogical effectiveness. By comparing this dataset with findings from other institutions or regions, researchers can identify trends or anomalies in AI adoption shielding light on contextual factors influencing acceptance. Moreover, the detailed profiles of respondents allow for segmentation analyses, exploring how different groups respond to AI tools, which can inform targeted training ang resources. Researchers may also use the data to conduct longitudinal studies, tracking changes in perceptions over time as AI technology evolves. This dataset can serve as a baseline for developing new survey instruments, enhancing reliability through established responses, thereby fostering ongoing research in the dynamic field of educational technology integration.

## 2 Background

The research on AI Copilot is anchored in theoretical frameworks that examine technology adoption within educational contexts. A central framework is the Technology Acceptance Model (TAM), which priotitizes perceived usefulness and ease of use as essential factors driving the acceptance of AI tools, including Copilot. This research expands on TAM by incorporating the AI Ecological Education Policy Framework, particularly its pedagogical dimension, to address ethical dilemmas and evolving curriculum design in education. The study leverages insights from [1], which focused on the adoption of generative AI in Hong Kong’s academic settings. As tools like AI Copilot gain traction globally, understanding the factors influencing their acceptance becomes imperative. By honing on the pedagogical dimension, the research aims to evaluate how both students and instructors perceive and adapt to AI integration in terms of curriculum assessment, skill development, and ethical considerations. This focused inquiry provides critical insights into the acceptance of AI technologies in education while addressing potential barriers, ensuring that educators and learners are well-equipped to navigate an increasingly AI-driven educational landscape.

## 3 Data Description

The dataset named AICopilotInEducation-dataset.xls consists of 49 columns representing survey items and 414 rows corresponding to individual responses. The first column records the start time of answering the questionnaire, while the second column captures the submission time. The third column contains the computed duration in minutes, calculated as the difference between the start and end times of answering the survey. The fifth column records the respondent’s consent to participate in the study. The demographic profile of the respondents is captured in columns six to twelve, which include information on age, gender, type of respondent (student or teacher), highest educational attainment, year level for student respondents, and years of AI usage. The summarized demographic profile of the respondents is presented in Table 1, Table 2, Table 3, Table 4, Table 5, Table 6, Table 7. Specifically, Table 1 shows the distribution of respondents according to age, while Table 2 presents the distribution based on gender. Table 3 summarizes the type of respondents, categorized as either student or teacher. Table 4 provides the respondents' highest educational attainment, and Table 5 shows the distribution according to college level for respondents enrolled in tertiary education. Meanwhile, Table 6 presents the year level of student respondents, and Table 7 summarizes the respondents’ years of AI usage. Table 8 displays the Items Codebook used in the data collection process. In addition, Table 9 presents the respondents' perceptions of the constructs under the pedagogical dimension, while Table 10 summarizes their perceptions of the constructs under the Technology Acceptance Model (TAM).

**Table 1:** Profile of the respondents in terms of age.

Category	Frequency	Percentage
18–24 years	374	94.44 %
25–34 years	12	3.03 %
35–44 years	6	1.52 %
45–54 years	3	0.76 %
55+ years	1	0.25 %

**Table 2:** Profile of the respondents in terms of gender.

Category	Frequency	Percentage
Female	200	50.51 %
Male	196	49.49 %

**Table 3:** Profile of the respondents in terms of type of respondent.

Category	Frequency	Percentage
Student	372	94 %
Teacher	24	6 %

**Table 4:** Profile of the respondents in terms of highest educational attainment.

Category	Frequency	Percentage
College Level	366	92.42 %
Bachelor’s Degree	16	4.04 %
Master’s Degree	8	2.02 %
Doctorate Degree (PhD. EdD)	6	1.52 %

**Table 5:** Profile of the respondents in terms of college.

Category	Frequency	Percentage
College of Computer, Information and Communications Technology	138	34.85 %
College of Technology	71	17.93 %
College of Education	67	16.92 %
College of Engineering	46	11.62 %
College of Arts and Sciences	35	8.84 %
College of Management and Entrepreneurship	34	8.59 %
College of Nursing	3	0.76 %
Others (such as College of Hospitality and tourism management and College of Customs)	2	0.51 %

**Table 6:** Profile of the respondents in terms of year level of student respondents.

Category	Frequency	Percentage
Second Year	194	53 %
First Year	94	25 %
Third Year	54	15 %
Fourth Year	27	7 %

**Table 7:** Profile of the respondents in terms of years of AI Usage.

Category	Frequency	Percentage
1–2 years	185	47 %
0–1 year	137	35 %
3–4 years	47	12 %
beyond 4 years	27	7 %

**Table 8:** Measurement item codebook.

Construct	Item	Description	Source
**Pedagogical:** Rethinking assessment and examinations (RAE)	RAE1	The assessment design should allow AI Copilot to enhance learning outcomes rather than solely producing output.	[[2], [3], [4]]
	RAE2	An assessment must focus on students’ comprehension to prevent AI Copilot-generated content from compromising the assessment process.	
	RAE3	Assessment must be automated scoring to speed up marking times and to reduce human bias.	
	RAE4	Use portfolio assessments to track students’ progress and show their skills across tasks.	
	RAE5	Use rubrics to set clear performance standards, allowing for fair and organized assessment of student work.	
**Pedagogical:** Developing student holistic competencies/generics skills(GS)	GS1	Teach the students to evaluate the credibility of AI Copilot-generated content.	[1,5,6]
	GS2	Create learning activities incorporating instructional strategies that enhance students’ critical thinking skills.	
	GS3	Create educational resources that would enhance students’ self-regulation and self-management.	
	GS4	Create active learning tasks that enhance students’ learning of social responsibility and ethical skills.	
	GS5	Allow the students to learn workplace skill.	
**Pedagogical:** Preparing students for the AI-driven workplace. (PS)	PS1	Teach the students how to use AI Copilot responsibly, ethically, and effectively.	[1,7,8]
	PS2	The university must develop curricula that reflect the increasing prominences of AI in various industries, ensuring that students are equipped with the skills and knowledge to navigate the evolving workplace landscape.	
	PS3	Curricula should integrate AI’ impact, emphasize ethics, and prioritize applied skills over rote memorization.	
	PS4	Measure key skills for human-AI collaboration by prioritizing portfolios over standardized scores to capture growth.	
	PS5	Prepare the students for life-long, student-driven learning.	
**Pedagogical:** Encouraging balanced approach to AI adoption (BA)	BA1	Incorporate AI Copilot in the development of assignments and assessments.	[1,9,10]
	BA2	Learn how AI Copilot can assist me, but that AI Copilot cannot replace me in doing my schoolwork.	
	BA3	Balance the automation provided by AI Copilot by understanding that I am still the pilot of my work.	
	BA4	Provide adequate training for users to enhance their skills and confidence in using AI Copilot.	
	BA5	The university must address ethical issues with AI Copilot, like bias, transparency, and mental health effects, to ensure it benefits all users, including those with special needs.	
**Perceived Usefulness(PU)**	PU1	Using AI Copilot in my work would enable me to accomplish tasks more quickly.	[[11], [12], [13]]
	PU2	Using AI Copilot can improve the efficiency of my work.	
	PU3	Advancing studies using AI Copilot can help me acquire the information I want.	
	PU4	Using AI Copilot in my job or learning would increase my productivity.	
	PU5	AI Copilot would improve my learning or job performance.	
**Perceived Ease of Use(PEOU)**	PEOU1	Learning to operate AI Copilot would be easy for me.	[[11], [12], [13]]
	PEOU2	My interaction with AI Copilot does not require much mental effort.	
	PEOU3	It is easy to become skillful at using AI Copilot.	
	PEOU4	I found AI Copilot easy to use.	
	PEOU5	It would be easy for me to find information using AI Copilot.	
**Intention to use AI Copilot (ITU)**	ITU1	I intend to use AI Copilot to a greater extent.	[14,12,13]
	ITU2	I think doing my schoolwork using AI Copilot is interesting.	
	ITU3	I believe that AI Copilot is a valuable tool for doing my schoolwork.	
	ITU4	I will recommend AI Copilot to another schoolmate or colleague.	
	ITU5	I believe doing my school work through AI Copilot has given me a unique experience.

**Table 9:** Perception on the construct of Pedagogical Dimension.

Item	Mean	Standard Deviation	Verbal Description
RAE1	3.65	1.05	Agree
RAE2	4.09	1.03	Agree
RAE3	3.42	1.05	Agree
RAE4	4.03	1.04	Agree
RAE5	4.28	1.05	Strongly Agree
GS1	3.93	1.06	Agree
GS2	4.26	1.01	Strongly Agree
GS3	3.67	1.00	Agree
GS4	4.20	0.98	Strongly Agree
GS5	4.38	0.98	Strongly Agree
PS1	4.17	1.08	Agree
PS2	3.77	1.01	Agree
PS3	4.03	1.05	Agree
PS4	3.82	0.98	Agree
PS5	4.30	1.00	Strongly Agree
BA1	3.61	0.99	Agree
BA2	3.90	0.99	Agree
BA3	4.15	1.04	Agree
BA4	3.89	1.05	Agree
BA5	4.13	1.04	Agree

Legend: 4.21–5.00 Strongly Agree; 3.41–4.20 Agree; 2.61–3.40 Neutral; 1.81–2.60 Disagree; 1.00–1.80 Strongly Disagree

**Table 10:** Perception on the constructs of TAM.

Item	Mean	Standard Deviation	Description
PU1	3.95	1.05	Agree
PU2	3.84	1.05	Agree
PU3	3.97	0.99	Agree
PU4	3.77	1.06	Agree
PU5	3.80	1.04	Agree
PEOU1	3.83	0.94	Agree
PEOU2	3.48	1.00	Agree
PEOU3	3.48	0.99	Agree
PEOU4	3.83	0.94	Agree
PEOU5	3.92	0.93	Agree
ITU1	3.66	0.99	Agree
ITU2	3.58	0.98	Agree
ITU3	3.70	0.94	Agree
ITU4	3.73	0.98	Agree
ITU5	3.72	0.98	Agree

Legend: 4.21–5.00 Strongly Agree; 3.41–4.20 Agree; 2.61–3.40 Neutral; 1.81–2.60 Disagree; 1.00–1.80 Strongly Disagree

## 4 Experimental Design, Materials and Methods

**Figure 1:**
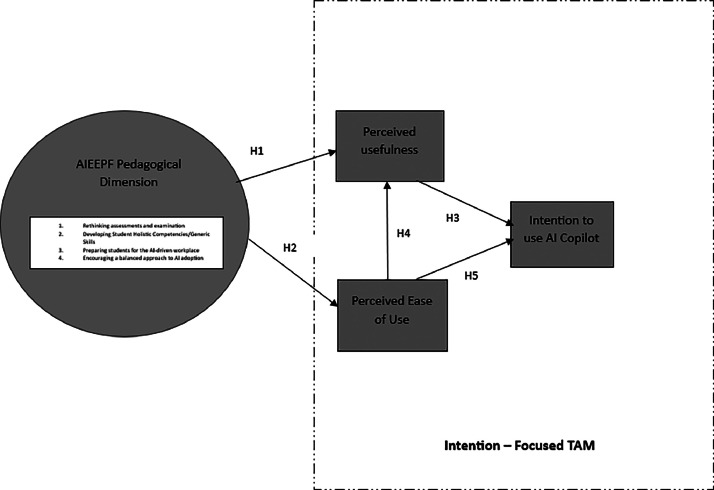
Research model.

A structured questionnaire was developed, incorporating key constructs from the Technology Acceptance Model (TAM) and the pedagogical dimension of the AI Ecological Education Policy Framework (AIEEPF), as illustrated in Figure 1, Research Model. The questionnaire items were derived from an extensive literature review and subsequently validated through indicator loadings, convergent validity, and reliability tests, as summarized in Table 11.

**Table 11:** Indicator loadings, convergent validity and reliability tests.

Constructs	Items	Factors Loadings	Cronbach’s α	CR	AVE
Pedagogical Dimension	RAE2	(0.779)	0.960	0.964	0.629
	RAE4	(0.714)			
	RAE5	(0.782)			
	GS1	(0.786)			
	GS2	(0.845)			
	GS4	(0.827)			
	GS5	(0.849)			
	PS1	(0.820)			
	PS2	(0.726)			
	PS3	(0.814)			
	PS4	(0.731)			
	PS5	(0.860)			
	BA2	(0.756)			
	BA3	(0.803)			
	BA4	(0.744)			
	BA5	(0.829)			
Perceived Usefulness	PU1	(0.846)	0.915	0.937	0.747
	PU2	(0.899)			
	PU3	(0.861)			
	PU4	(0.847)			
	PU5	(0.867)			
Perceived Ease of Use	PEOU1	(0.834)	0.859	0.905	0.704
	PEOU3	(0.783)			
	PEOU4	(0.880)			
	PEOU5	(0.856)			
Intention to Use AI Copilot	ITU1	(0.812)	0.899	0.925	0.713
	ITU2	(0.817)			
	ITU3	(0.869)			
	ITU4	(0.881)			
	ITU5	(0.840)			

In Structural Equation Modeling (SEM), assessing the measurement model is crucial to establishing the validity and reliability of the constructs. Key metrics include factor loadings, Cronbach’s α, Composite Reliability (CR), and Average Variance Extracted (AVE). Factor loadings measure the strength of the relationship between observed variables and their latent constructs, with values above 0.70 indicating strong indicators, thus supporting convergent validity [15]. AVE values of 0.50 or higher further confirm that at least half of the variance in the indicators is explained by the construct [15].

Cronbach’s α assesses internal consistency, with a commonly accepted threshold of 0.70 or higher; however, values between 0.60 and 0.70 may be acceptable in certain research contexts [16]. This reliability measure ensures the consistency of the data collection instrument, as highlighted in previous studies [17,18]. Similarly, Composite Reliability (CR) values of 0.70 or above are acceptable, while values exceeding 0.80 indicate excellent reliability of the latent constructs [18].

Collectively, these metrics—Factor Loadings, Cronbach’s α, CR, and AVE—offer a comprehensive framework for evaluating measurement models in SEM. Ensuring both reliability and validity enhances the quality, accuracy, and interpretability of research findings derived through SEM methodologies [15,18].

A non-probability convenience sampling method was utilized, targeting students and instructors within Cebu Technological University (CTU). The survey was distributed electronically using Google Forms to ensure accessibility and convenience for respondents.

Table 12 presents the assessment of Discriminant Validity using the Fornell-Larcker Criterion, a widely accepted method in SEM. This criterion requires that the square root of the AVE for each construct exceed its correlations with other constructs, confirming construct distinctiveness and validity. Scholars underscore the importance of this criterion in SEM [15,19], emphasizing its role in ensuring both statistical and conceptual uniqueness of constructs.

**Table 12:** Discriminant validity using the Forknell-Larcker Criterion.

Constructs	1	2	3	4
1. Pedagogical Dimension	(0.793)	0.719	0.640	0.628
2. Perceived Usefulness	0.719	(0.864)	0.700	0.752
3. Perceived Ease of Use	0.640	0.700	(0.839)	0.773
4. Intention to Use AI Copilot	0.628	0.752	0.773	(0.844)

Furthermore, Table 13 illustrates Discriminant Validity using the Heterotrait-Monotrait Ratio of Correlation (HTMT), another critical technique in SEM. The HTMT ratio helps determine if constructs are sufficiently distinct, with a common threshold of 0.85, and a stricter threshold of 0.90 used in some cases [20]. Table 14 presents the Model Fit and Quality Indices. Table 15 summarizes the Coefficient of Determination (R²), Full Collinearity VIF, and Stone-Geisser’s Q² values for endogenous constructs, offering additional validation of model reliability and predictive accuracy.

**Table 13:** Discriminant validity using HTMT ratio of correlations.

Constructs	1. Pedagogical Dimension	2	3
2. Perceived Usefulness	0.769		
3. Perceived Ease of Use	0.703	0.789	
4. Intention to Use AI Copilot	0.680	0.829	0.880

**Table 14:** Model fit and quality indices.

Index Name	Values	Criterion (Kock, 2020)
Average Path Coefficient (APC)	0.480, P<0.001	P <0.05
Average R-Squared (ARS)	0.573, P<0.001	P <0.05
Average Adjusted R-Squared (AARS)	0.571, P<0.001	P<0.05
Average block VIF (AVIF)	1.917	Acceptable if <= 5, ideally <=3.3
Average Collinearity VIF (AFVIF)	2.832	Acceptable if <= 5, ideally <=3.3
Tenenhaus GOF	0.632	Small >=0.1; mediam>=0.25; large >=0.36
Simpson’s Paradox Ratio (SPR)	1.000	Acceptable if >=0.7, ideally =1
R-Squared contribution Ratio (RSCR)	1.000	Acceptable if >=0.9, ideally = 1
Statistical Suppression Ratio (SSR)	1.000	Acceptable if >=0.7
Nonlinear Bivariate Causality Direction Ratio	1.000	Acceptable if >=0.7

**Table 15:** Coefficient of determination, full collinearity VIF, Q2.

Endogenous Construct	R^2^	Full Collinearity VIF	Q^2^
1. Perceived Usefulness	0.614	3.076	0.614
2. Perceived Ease of Use	0.420	2.826	0.419
3. Intention to Use AI Copilot	0.683	3.181	0.682

**Figure 2:**
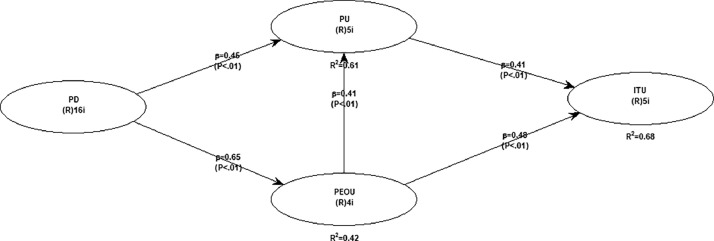
Structural model with beta coefficient.

The results of hypothesis testing are presented in Table 16, providing crucial insights into the relationships between variables within the proposed model. Figure 2 graphically illustrates the tested research model and the structural relationships among the constructs.

**Table 16:** Results of hypothesis testing.

Hypothesis	Path	Β	P-value	f^2^	Decision
Direct Relationship					
1	PD→PU	0.446	<0.001	0.322	Supported
2	PD→PEOU	0.648	<0.001	0.420	Supported
3	PU→ITU	0.415	<0.001	0.313	Supported
4	PEOU→PU	0.412	<0.001	0.293	Supported
5	PEOU→ITU	0.478	<0.001	0.370	Supported
Indirect Effect					
6	PD→PEOU→PU	0.267	<0.001	0.193	Supported
7	PEOU→PU→ITU	0.171	<0.001	0.132	Supported

Data analysis was performed using WarpPLS 8.0, a software specifically designed for Partial Least Squares Structural Equation Modeling (PLS-SEM). WarpPLS facilitates the evaluation of both the measurement model and the structural model, providing robust insights into the interrelationships among constructs within the study.

## Limitations

The study on AI Copilot adoption at Cebu Technological University faced several data-related limitations. Initially, 414 responses were collected, but 18 were excluded due to minimal variability, which reduced the sample size to 396 and may have led to the omission of potentially valuable insights. The convenience sampling method may also have introduced bias, as the respondents likely consisted of individuals with a greater interest in technology, thus skewing representation. Morever, relying on self-reported data presents challenges, responses could be affected by social desirability bias or misunderstandings of the survey questions.

## Ethics Statement

Survey participants provided informed consent before completing the survey and recorded their responses anonymously. No personally identifiable data was collected or retained. Ethical approval from the institutional board was not required for this study.

## CRediT Author Statement

**Emelie C. Villaceran:** Conceptualization, Methodology, Investigation, Writing – Original Draft

**Celbert M. Himang:** Software, Validation, Formal Analysis, Visualization, Supervision

## Data Availability

Mendeley DataData on Behavioural Intention to Use AI Copilot Through TAM and AI Ecological Policy Lens (Original data)
